# Factors Associated With Penicillin Allergy Labels in Electronic Health Records of Children in 2 Large US Pediatric Primary Care Networks

**DOI:** 10.1001/jamanetworkopen.2022.2117

**Published:** 2022-03-14

**Authors:** Margaret G. Taylor, Torsten Joerger, Yun Li, Michael E. Scheurer, Michael E. Russo, Jeffrey S. Gerber, Debra L. Palazzi

**Affiliations:** 1 Division of Infectious Diseases, Department of Pediatrics, Baylor College of Medicine, Texas Children’s Hospital, Houston; 2 Division of Infectious Diseases, Children’s Hospital of Philadelphia, Department of Pediatrics, Perelman School of Medicine, University of Pennsylvania, Philadelphia; 3 Now with Division of Infectious Diseases, Department of Pediatrics, Stanford University School of Medicine and Lucile Packard Children’s Hospital Stanford, Stanford; 4 Department of Biostatistics, Epidemiology, and Informatics, Perelman School of Medicine, University of Pennsylvania, Philadelphia; 5 Department of Pediatrics, Perelman School of Medicine, University of Pennsylvania, Philadelphia; 6 Pediatric IDEAS Research Group of the Center for Pediatric Clinical Effectiveness, Children’s, Phildelphia; 7 Division of Hematology-Oncology, Department of Pediatrics, Baylor College of Medicine, Texas Children’s Hospital, Houston

## Abstract

**Importance:**

Penicillin allergy labels influence clinical decision-making, yet most children who are labeled do not have type 1 hypersensitivity allergic reactions and instead have a history of predictable adverse reactions or unspecified illness symptoms while receiving penicillin for viral infections. Studies describing penicillin allergy labeling in the pediatric outpatient setting are lacking.

**Objective:**

To describe the epidemiology and factors associated with penicillin allergy labels across 2 large US pediatric primary care networks.

**Design, Setting, and Participants:**

This retrospective, longitudinal birth cohort study was conducted in 90 primary care pediatric practices serving a diverse population of children across Houston, Texas, Austin, Texas, Philadelphia, Pennsylvania, and parts of New Jersey. Participants were children born between January 2010 and June 2020 who had a health care visit in the first 14 days of life and at least 2 additional visits in the first year of life at one of 90 primary care pediatric practices. Censoring criteria were additionally applied to exclude data from children no longer seeking health care in the 90 clinics over time. Statistical analysis was performed from February to May 2021.

**Exposures:**

Basic patient demographics, health care utilization, penicillin exposure, and primary clinic location.

**Main Outcomes and Measures:**

Addition of penicillin allergy label in the electronic medical record.

**Results:**

Among 334 465 children in the birth cohort, 164 173 (49.1%) were female; 72 831 (21.8%) were Hispanic, 59 598 (17.8%) were non-Hispanic Black, and 148 534 (44.4%) were non-Hispanic White; the median (IQR) age at censoring was 3.8 (1.7-6.6) years; 18 015 (5.4%) were labeled as penicillin allergic, but the prevalence of penicillin allergy labeling ranged from 0.9% to 10.2% across practices. Children were labeled at a median (IQR) age of 1.3 (0.9-2.3) years. Non-Hispanic White children were more likely to be labeled compared with non-Hispanic Black children after controlling for potential confounders (adjusted odds ratio, 1.7 [95% CI, 1.6-1.8]). There were 6797 allergic children (37.7%) labeled after receiving 1 penicillin prescription and 1423 (7.9%) labeled after receiving 0 penicillin prescriptions.

**Conclusions and Relevance:**

In this cohort study of more than 330 000 children, penicillin allergy labeling was common and varied widely across practices. Children were labeled early in life, and almost half were labeled after receiving 1 or 0 penicillin prescriptions. These findings raise questions regarding the validity of penicillin allergy labels. Future work exploring the fidelity of and outcomes associated with penicillin allergy–labeling in children is warranted.

## Key Points

**Question:**

What factors are associated with penicillin allergy labeling in the pediatric primary care setting?

**Findings:**

In this birth cohort study of 334 465 children at 90 primary care pediatric practices, non-Hispanic White ethnicity and race was associated with a penicillin allergy label. Most children carrying penicillin allergy labels were labeled before 2 years of age, and many were labeled after receiving 0 or 1 penicillin prescriptions; allergy labeling varied widely across practices.

**Meaning:**

These findings raise questions regarding the validity of penicillin allergy labels among pediatric outpatients and highlight the importance of and potential targets for allergy labeling stewardship in this population to help curb unnecessary use of second-line antibiotics.

## Teaser

This cohort study examines the epidemiology and factors associated with penicillin allergy labels among pediatric patients across 2 large US pediatric primary care networks.

## Introduction

Penicillin allergy is the most common drug allergy in the world.^1,2,3,4,5^ Although 5% to 10% of the population is labeled as penicillin allergic,^6,7,8,9^ more than 95% of children with this allergy label are not found to have a type 1 IgE-mediated hypersensitivity after penicillin skin testing or oral challenge.^4,10,11,12,13,14^ Unconfirmed penicillin allergy labels (PALs), specific designations in the medical record to signify a history of penicillin allergy, can lead to potentially unnecessary receipt of broad spectrum, second-line antibiotics. In the hospital setting, children with unconfirmed PALs have prolonged hospital stays, higher rates of adverse events, and more infections due to *Clostridium difficile* and vancomycin-resistant *Enterococcus* compared with children without PALs.^15,16,17,18,19,20^ Despite the potential harms associated with unsubstantiated PALs, allergy labels are often left unevaluated in a child’s electronic health record (EHR).^21^

In 2016, the Infectious Disease Society of America and the Society for Healthcare Epidemiology of America identified PALs as a priority for antimicrobial stewardship.^22^ Subsequent initiatives have been implemented in the inpatient and emergency department settings to address PALs.^23,24,25^ However, many children are never seen in these settings, and most antibiotics are prescribed in the primary care outpatient setting.^26^ Additionally, children with common outpatient infections may be evaluated by a medical professional other than their primary care clinician during times of illness, making PAL supervision challenging during acute care visits. Preventative health care visits with a child’s primary care physician (PCP) offers an optimal medical home for addressing PALs with a parent-trusted clinician who has a working relationship with a family. However, the epidemiology, mechanisms of penicillin allergy labeling, and the best way to address PALs in this setting are unclear. A better understanding of the factors that lead to the placement of a PAL can inform penicillin allergy delabeling efforts for children who are not truly allergic and prevent inappropriate allergy labeling. Toward this end, we explored the epidemiology and factors associated with PALs across 2 of the largest pediatric primary care networks in the United States: Texas Children’s Pediatrics (TCP) and Children’s Hospital of Philadelphia (CHOP).

## Methods

### Study Design

This was a dual-center, retrospective, longitudinal birth cohort study. The Baylor College of Medicine and CHOP Institutional Review Boards for the Protection of Human Subjects approved the study and granted a waiver of informed consent. This report followed the Strengthening the Reporting of Observational Studies in Epidemiology (STROBE) reporting guideline.^27^

### Setting

TCP and CHOP incorporate 90 hospital-affiliated pediatric primary care centers that employ more than 500 board-certified pediatricians and advanced registered nurse practitioners in Houston and Austin, Texas (TCP) and Pennsylvania and New Jersey (CHOP). In 2019 and 2020, more than 700 000 children were evaluated in more than 3.7 million encounters at TCP and CHOP primary care clinics. Clinicians within both settings use an EHR system called EpiCare (Epic Systems Inc) for office and telephone encounter documentation and order entry.

### Study Population

Children were included in the birth cohort if they (1) were born between January 1, 2010, and June 30, 2020; (2) were seen in-person or via telehealth with any TCP or CHOP PCP within the first 14 days of life, and (3) completed at least 2 additional PCP visits in the first year of life. Data were censored on the date of a child’s last qualifying PCP visit, including at least 1 PCP encounter per year for ages 1 to 4 years and 1 PCP encounter every 2 years after 4 years of age.

### Variables

Patient-level data extracted from the EHR included sex, age, race, ethnicity, primary language, and problem list *International Statistical Classification of Diseases and Related Health Problems, Tenth Revision (ICD-10)* codes at the censor date or date of data extraction (November 12, 2020, for TCP; December 13, 2020, for CHOP). Ethnicity and race data were self-reported and classified into Asian or Pacific Islander, Hispanic, non-Hispanic Black, non-Hispanic White, or other ethnicity or race. Chronic illness was defined from a patient’s problem list using the pediatric complex chronic conditions classification system version 2.^28^ Visit-level data extracted from the EHR included practice location, encounter date, encounter type (health care visit [appointment, office visit, e-visit, telemedicine appointment, telemed-remote site, or mobile app encounter] or communication [telephone encounter, patient message, or patient outreach]), clinician name, encounter *ICD-10* codes, and insurance used. Children with at least 1 encounter covered by government insurance were further classified as having government insurance. Patients were assigned to a primary clinic based on the location of their first completed primary care office visit. Medications were captured from the EHR in all health care settings associated with Texas Children’s Hospital (TCH, including TCP clinics) and CHOP, including primary care clinics, urgent care centers, specialty clinics, emergency departments, and hospitals. Allergy data extracted from the EHR included name of allergen and date of penicillin allergy label placement and removal (if applicable). Electronically extracted data were validated using manual medical record review in each health system’s EHR.

### Outcome Variables

The primary outcome was addition of a PAL in the EHR, defined as an allergy label to a penicillin derivative (including penicillin, ampicillin, amoxicillin, piperacillin-tazobactam, amoxicillin-clavulanate, ampicillin-sulbactam, or antistaphylococcal penicillin). Children with isolated cephalosporin or carbapenem allergies were not included in the PAL group.

### Statistical Analysis

Descriptive statistics were expressed as frequencies for categorical variables and as medians with interquartile ranges (IQR, 25th and 75th percentiles) for continuous variables. Factors associated with PALs were explored using mixed-effect logistic regression, accounting for patient clustering among primary clinics. Patient-specific covariates were selected a priori based on clinical knowledge and relevance of their association with penicillin allergy labeling and included sex, race, primary language, chronic condition, age, payer status, health care utilization, and penicillin exposure. Health care utilization was defined as total primary care visits and communications by 2 years of age, and penicillin exposure was defined as receipt of a penicillin prescription from a primary care physician in the first 2 years of life. The cutoff of 2 years was selected based on initial analyses showing most PALs and penicillin prescriptions occurred before 2 years of age.

The amount of total variation in log odds of having a PALs across the primary care clinics (A) was calculated using a mixed-effect logistic regression without any covariates. For any covariate of interest, we included it in the model to obtain residual variation in log odds of having a PAL across the primary care clinics (B). The percent of variation explained by this covariate was calculated as (A-B)/A. We calculated the percentage of variation in log odds of having a PAL across clinics explained by covariates of interest independently and jointly.

All analyses were performed using Stata software 16 (StataCorp) from February to May 2021. A 2-sided 5% significance level (*P* < .05) was used for all statistical inferences.

## Results

### Birth Cohort

A total of 334 465 children were included in the birth cohort (206 451 at TCP and 128 014 at CHOP) (Table 1); 164 173 (49.1%) were female; 72 831 (21.8%) were Hispanic, 59 598 (17.8%) were non-Hispanic Black, and 148 534 (44.4%) were non-Hispanic White; the median (IQR) age at censor date was 3.8 (1.7-6.6) years. A total of 1.4 million person-years were captured during the study period. There were more Hispanic children in the TCP network (60 301 [29.2%]) and more non-Hispanic Black children in the CHOP network (33 208 [25.9]) (Table 1).

**Table 1.** Basic Demographics, Health Care Encounters, and Penicillin Prescriptions Captured in a 10-Year Retrospective Dual-Center Birth Cohort

Characteristic	Patients, No. (%)		
	Combined centers (N = 334 465)	Texas Children’s Pediatrics (n = 206 451 [61.7%])	Children’s Hospital of Philadelphia (n = 128 014 [38.3%])
Sex			
Female	164 173 (49.1)	101 601 (49.2)	62 572 (48.9)
Male	170 292 (50.9)	104 850 (50.8)	65 442 (51.1)
Ethnicity and race			
Asian or Pacific Islander	21 081 (6.3)	14 546 (7.0)	6535 (5.1)
Hispanic	72 831 (21.8)	60 301 (29.2)	12 530 (9.8)
Non-Hispanic Black	59 598 (17.8)	26 390 (12.8)	33 208 (25.9)
Non-Hispanic White	148 534 (44.4)	86 008 (41.7)	62 526 (48.8)
Other^a^	32 421 (9.7)	19 206 (9.3)	13 215 (10.3)
Primary language			
English	309 828 (92.6)	193 978 (94.0)	115 850 (90.5)
Spanish	8926 (2.7)	6511 (3.2)	2415 (1.9)
Other	2499 (0.7)	1092 (0.5)	1407 (1.1)
Missing	13 212 (4.0)	4870 (2.4)	8342 (6.5)
Insurance type			
Government	119 988 (35.9)	64 147 (31.1)	55 841 (42.6)
Private	214 477 (64.1)	142 304 (68.9)	72 173 (57.4)
Chronic condition	24 571 (7.4)	14 717 (7.1)	9854 (7.7)
Age, median (IQR), y	3.8 (1.7-6.6)	3.8 (1.7-6.6)	5.3 (2.8-8.0)
Total primary care encounters	12 205 235	6 693 537	5 511 698
Primary care health care visits	6 999 373 (57.3)	4 340 318 (64.8)	2 659 055 (48.2)
Primary care communications	5 205 862 (42.7)	2 353 219 (35.2)	2 852 643 (51.8)
Primary care penicillin prescriptions	759 616	492 169	267 447

^a^ Other ethnicity and race included American Indian, Alaskan Native, and self-reported other.

Among approximately 12.2 million primary care health care encounters, 6 999 373 (57.3%) were in-person or telemedicine visits. There were 6 395 679 patient encounters (91.3%) that occurred at a child’s assigned primary care clinic. A total of 946 592 penicillin derivatives were prescribed during the study period; 759 616 (80.2%) were prescribed in the primary care setting (Table 1) and 186 976 (19.8%) were prescribed elsewhere, including emergency care centers, urgent care centers, hospitals, and subspecialty clinics. There were differences in rates of penicillin prescribing by 2 years of age among children of different racial and ethnic backgrounds (eTable 1 in the Supplement). Most penicillin derivatives in the primary care setting were narrow-spectrum amoxicillin or penicillin (n = 606 317; 79.8%), whereas the rest were a beta lactam plus beta lactamase inhibitor (n = 153 299; 20.2%).

### Penicillin Allergy Labeling

**Figure 1.**
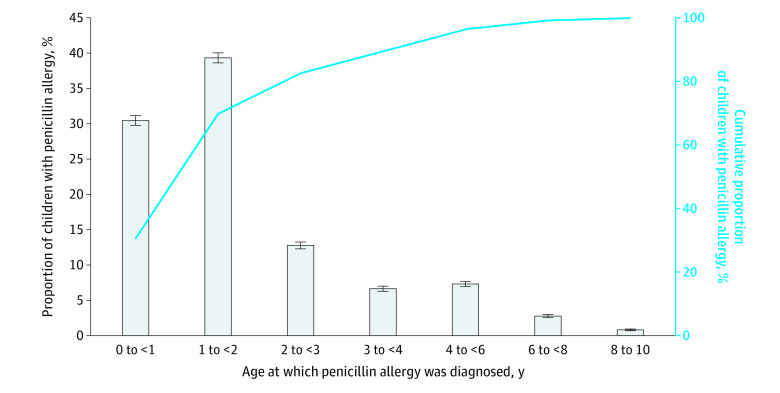
Proportion of Children Labeled as Penicillin Allergic by Age Bar graph (left axis) proportion of children labeled in each age group. Line graph (right axis) cumulative proportion of children labeled as penicillin allergic in birth cohort. Error bars indicate 95% CIs.

In the birth cohort, 18 015 children (5.4%) were labeled as penicillin allergic (Table 2). Most PALs (n = 16 354 [90.8%]) were placed within 24 hours of a primary care encounter or health care communication, but only 12.5% of PALs (n = 2244) were eventually removed from the EHR during the study period. The median (IQR) age of children labeled as penicillin allergic was 1.3 (0.9-2.3) years, and 16 069 (89.2%) were labeled by 4 years of age (Figure 1). A subanalysis of children who were labeled and remained in the birth cohort at least 4 years found similar results in age of PAL placement (eFigure 1 in the Supplement). Non-Hispanic White children made up a larger percentage of the group labeled as penicillin allergic compared with the group without PALs (Table 2; eTable 2 in the Supplement).

**Table 2.** Patient Demographics of Children Labeled as Penicillin Allergic and Nonallergic in a Dual-Center Birth Cohort of 334 465 Individuals

Characteristic	Penicillin allergic (n = 18 015 [(5.4%])	Penicillin nonallergic (n = 316 450 [94.6%])
Sex, No. (%)		
Female	8331 (46.2)	155 842 (49.2)
Male	9684 (53.8)	160 608 (50.8)
Ethnicity and race, No. (%)		
Asian or Pacific Islander	1009 (5.6)	20 272 (6.3)
Hispanic	3234 (18.0)	69 597 (22.0)
Non-Hispanic Black	1651 (9.2)	57 947 (18.3)
Non-Hispanic White	10 689 (59.3)	137 845 (43.6)
Other^a^	1432 (7.9)	30 989 (9.8)
Primary language, No. (%)		
English	17 110 (95.0)	292 713 (92.5)
Spanish	372 (2.1)	8554 (2.7)
Other	108 (0.6)	2391 (0.8)
Missing	425 (2.4)	12 787 (4.0)
Government insurance, No. (%)	4870 (27.0)	115 118 (36.4)
Chronic condition, No. (%)	1319 (7.3)	23 252 (7.4)
Age, median (IQR), y	5.7 (3.5-8.1)	3.6 (1.6-6.5)

^a^ Other ethnicity and race included American Indian, Alaskan Native, and self-reported other.

### Factors Associated With Penicillin Allergy Labeling

Of the 18 015 children with PALs, 6797 (37.7%) were labeled after their first penicillin prescription from any TCH or CHOP health care setting, and 1423 (7.9%) were labeled despite not having received any penicillin prescriptions (eFigure 2 in the Supplement).

In a mixed-effect logistic regression analysis, after adjusting for health care utilization, primary care penicillin exposure, and basic patient demographics, children identified as non-Hispanic White had significantly higher odds of being labeled as penicillin allergic compared with non-Hispanic Black children (adjusted odds ratio [aOR], 1.7 [95% CI, 1.6-1.8]) (Table 3). Children who received at least 1 primary care penicillin prescription by 2 years of age were more likely to be labeled compared with those who did not (aOR, 1.7 [95% CI 1.6-1.8] Table 3). Chronic condition and the patient’s documented primary language were not associated with penicillin allergy labeling.

**Table 3.** Results From a Univariable and Multivariable Mixed-Effect Logistic Regression Model for Penicillin Allergy Labeling, Accounting for Clustering of Patients Among Primary Clinics

Characteristic	Univariable analysis OR (95% CI)	*P* value	Multivariable analysis OR (95% CI)^a^	*P* value
Sex				
Female	1 [Reference]	NA	1 [Reference]	NA
Male	1.13 (1.10-1.16)	<.001	1.06 (1.02-1.09)	.001
Ethnicity and Race				
Non-Hispanic Black	1 [Reference]	NA	1 [Reference]	NA
Asian or Pacific Islander	1.45 (1.20-1.56)	<.001	1.46 (1.32-1.59)	<.001
Hispanic	1.45 (1.35-1.55)	<.001	1.40 (1.30-1.49)	<.001
Non-Hispanic White	2.11 (1.99-2.45)	<.001	1.69 (1.59-1.80)	<.001
Primary language				
English	1 [Reference]	NA	1 [Reference]	NA
Spanish	0.86 (0.77-0.96)	.007	0.98 (0.87-1.10)	.73
Other	0.79 (0.65-0.96)	.02	0.99 (0.83-1.23)	.93
Government insurance	0.80 (0.77-0.83)	<.001	0.94 (0.90-0.97)	.001
Chronic condition	1.09 (1.02-1.15)	.005	0.96 (0.90-1.02)	.15
By age 2 y				
Healthcare visits	1.09 (1.09-1.09)	<.001	1.05 (1.05-1.05)	<.001
Healthcare communications	1.03 (1.03-1.03)	<.001	1.01 (1.01-1.01)	<.001
Given penicillin	3.27 (3.15-3.39)	<.001	1.67 (1.61-1.75)	<.001

Abbreviations: NA, not applicable; OR, odds ratio.

^a^ Additionally adjusted for years in the birth cohort.

### Clinic Variation

**Figure 2.**
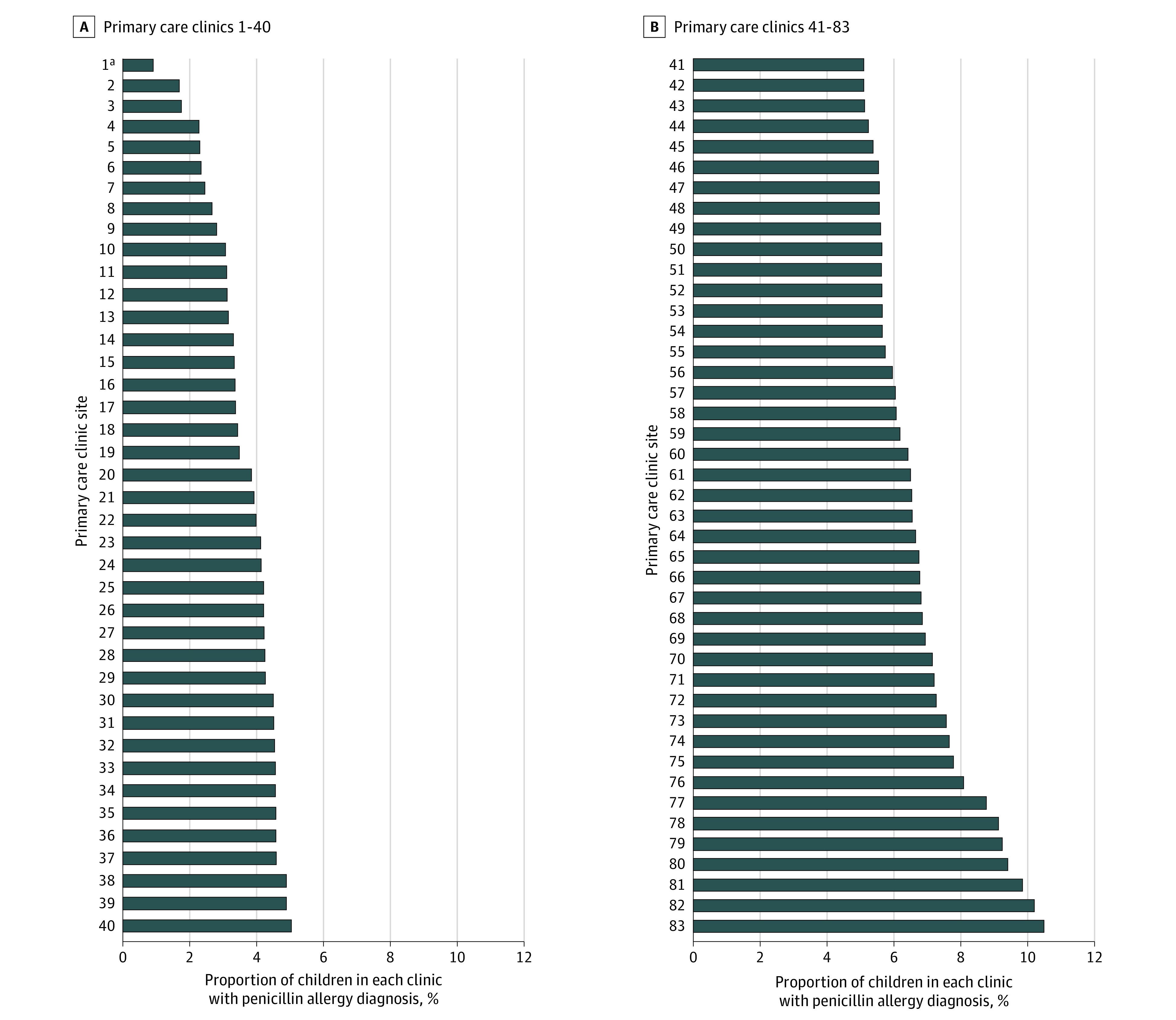
Variation in Prevalence of Penicillin Allergy Labels Among 90 Outpatient Primary Care Clinics ^a^This bar represents the average from 7 small primary care TCP clinics that had recently opened in Austin, Texas. All other bars in the figure represent a unique primary care clinic.

The unadjusted rates of penicillin allergy labeling at the primary care clinics ranged from 0.9% to 10.5% (Figure 2), but 83.5% of the variation was accounted for by differences in patient demographics, age, health care utilization, and penicillin exposure by 2 years of age (eTable 3 in the Supplement).

## Discussion

In a 10-year birth cohort of more than 330 000 children in 2 large pediatric primary care networks, penicillin allergy labeling was common and varied widely across practices. Children identified as non-Hispanic White were more likely to receive a PAL compared with non-Hispanic Black children, even after adjusting for potential confounders. Children were labeled young, and almost half of children were labeled after receiving 1 or 0 penicillin prescriptions, raising questions regarding the validity of PALs in this setting.

Prior studies have shown that less than 5% of children labeled as penicillin allergic have a type 1 hypersensitivity allergy on skin testing or oral challenge.^4,10,11,12,13,14^ If applied to this birth cohort, more than 16 000 children in the Houston and Philadelphia metropolitan regions likely have inaccurate PALs. Efforts to remove unconfirmed PALs have been described in the inpatient setting^22,23,24^ but an estimated 36% to 49% of patients with negative penicillin allergy testing will have persistence or redocumentation of their PALs in the primary care setting.^29,30^ In our study, few PALs were removed during the study period and the vast majority were placed within 24 hours of a PCP health care visit or telephone encounter. Therefore, quality improvement efforts to address unconfirmed penicillin allergy labels should engage directly with PCPs, who are most likely to interact with children outside of an acute illness and identify ways to prevent redocumenting PALs in children who have been cleared of their label.

Most IgE-mediated hypersensitivity reactions are thought to require repeated exposure to a hapten following an initial sensitization phase; therefore, in most cases, at least 2 distinct exposures to a penicillin derivative are required to elicit this hypersensitivity response.^31^ Previous reports have described that frequent or repetitive courses of penicillin are a major risk factor for PALs.^32^ However, in this study, 37.7% of children were labeled after their first penicillin prescription, suggesting that many pediatric PALs represent a prior anticipated adverse drug reaction or an unspecified viral illness. In one study of more than 5000 adults with a history of known adverse drug reaction to penicillin, 95% were incorrectly documented as an allergy rather than drug intolerance.^33^ Future work should explore the quality of penicillin allergy labels in children, particularly in children labeled after their first penicillin prescription. Additionally, even though many PALs are likely inconsistent with a true type 1 hypersensitivity reaction, many children will still require a graded oral challenge to remove a PAL. Therefore, quality improvement initiatives should focus on preventing inappropriate penicillin allergy labeling in addition to removing inaccurate PALs.

We found that 7.9% of children were labeled as penicillin allergic after having received 0 penicillin prescriptions in any TCP or CHOP setting. Although we could not exclude that these children received prescriptions from other health care systems, the use of a birth cohort with strict censoring criteria was used to minimize this limitation. Alternative hypotheses to explain the presence of a PAL without prior penicillin exposure include a possible exposure in utero to prenatal penicillin derivates (including intrapartum penicillin for group B streptococcus colonization), exposure to cephalosporin antibiotics (with theoretical cross-reactivity prompting documenting of a PAL), or a family history of penicillin allergy. Future work should explore the reasons for PAL documentation in children without a known penicillin exposure.

Most children were labeled as penicillin allergic by 4 years of age. Similar findings were described in a study in a pediatric emergency center, in which most children with penicillin allergy were labeled by age 3 years.^34^ Children in this age group are frequently diagnosed with upper respiratory tract infections, many of which are viral in etiology. Quality improvement efforts should consider targeting a young age group (<4 years of age) to address unconfirmed PALs before they become perpetuated into adolescence.

We found that rates of penicillin allergy labeling varied widely across clinics. Prior studies have shown variation in rates of accurate penicillin allergy labeling among different clinicians.^33,35^ Future work should explore this variation further, as identifying clinics with high PAL prevalence for allergy delabeling quality improvement initiatives will be an important first step for outpatient allergy stewardship. Future studies should also examine the quality of PALs based on who placed the label, particularly when labels are added outside of a child’s medical home or established primary care clinician. Finally, it would be interesting to see if rates of referral to allergy specialists varies based on who placed the allergy label, or if the presence of multiple antibiotic allergies prompts clinicians to refer to an allergist sooner than a single PAL

We observed higher rates of PALs among children identified as non-Hispanic White compared with other ethnicities or races. Similar differences have been suggested, but not fully explored, in other studies. One study found a lower prevalence of beta lactam allergies among Hispanic and Black patients vs non-Hispanic White patients.^2^ Another study described lower rates of PALs prior to penicillin skin test in Black vs White patients.^14^ In cases of asthma and atopy, social inequalities and access to care have been shown to contribute to disease severity and recurrence.^36,37^ However, race, as a social construct, is not known to be associated with genotypic risk for having a true type 1 hypersensitivity penicillin reaction.^37^ These racial differences in PAL frequency in this study persisted despite adjustment for primary clinic location, primary language, insurance status, health care utilization (including both health care visits and telephone communications), and penicillin exposure. One hypothesis for the persistent variation in PALs might include differential rash identification among children with different skin tones, which has been reported in children with Lyme disease.^38^ Alternative hypotheses might include more subtle disparities in access to care or different antibiotic prescribing practices or diagnostic thresholds for PALs among clinicians taking care of different racial groups.^39,40,41^ We also noted slightly increased rates of penicillin exposure by 2 years of age in non-Hispanic White children compared with non-Hispanic Black and Asian/Pacific Islander children, which is consistent with prior literature reports.^40^ However, even after accounting for these differences in the mixed-effect logistic regression analysis, there were higher odds of PAL placement in non-Hispanic White children. As the downstream effect of these PALs is the use of alternative, often second-line antibiotics to treat common pediatric infectious diseases, these differences merit additional investigation.

### Strengths and Limitations

This study has notable strengths. Most published pediatric studies examining penicillin allergy labeling have been limited to a few hundred children referred to allergy clinics, whereas this study examined penicillin allergy labeling in more than 330 000 children in the primary care setting. The population studied was racially, ethnically, socioeconomically, and geographically diverse, strengthening generalizability. Additionally, use of a birth cohort helped maximize complete capture of care and penicillin prescriptions during the study period.

There are limitations to this study. First, because EHR data from the TCP network began in 2010, the oldest children in the birth cohort were 10 years of age. However, in a subanalysis of children with PALs at CHOP beginning in 2004, fewer than 5% of children were labeled after 10 years of age. Second, because our birth cohort required children to have a primary care visit in the first 14 days of life and periodic subsequent in-person encounters, the results may not be generalizable to children who had substantial illness at birth or consistent follow-up with a PCP. Third, data were limited to documentation within the constructs of the EHR platform. Finally, we could not exclude the possibility that children received penicillin prescriptions in other health care settings outside TCP or CHOP, leading to an overestimation of the number of children who were labeled as penicillin allergic after receiving 0 penicillin prescriptions. However, the use of a birth cohort with strict censoring criteria and the inclusion of penicillin antibiotic prescriptions from all TCH and CHOP sites, was used to minimize the possibility of uncaptured penicillin prescriptions.

## Conclusions

In this 90-clinic pediatric birth cohort, including more than 500 clinicians and 330 000 patients, 5.4% of children were labeled as penicillin allergic with wide variation across clinics. Children of non-Hispanic White ethnicity and race were more likely to be labeled as allergic. Children were labeled young, and many children labeled as penicillin allergic received 1 or 0 penicillin prescriptions prior to PAL placement, raising questions regarding the validity of PALs in this setting. Future work exploring the fidelity of and outcomes associated with penicillin allergy labeling in children is warranted.

## Notes

Supplement. **eFigure 1.** Proportion of Children Labeled as Penicillin Allergic by Age in a Sub-population of Children Remaining in the Birth Cohort Greater Than 4 Years **eFigure 2.** Proportion of Children Labeled as Penicillin Allergic by the Total Number of Penicillin Prescriptions They Received Prior to Penicillin Allergy Label Placement **eTable 1.** Proportion of Children Receiving Primary Care Penicillin Prescriptions Before Two Years of Age by Race and Ethnicity **eTable 2.** Patient Demographics Among Penicillin Allergic and Non-Allergic Children by Primary Care Network **eTable 3.** Percent of Variation in Penicillin Allergy Label Prevalence Among the Primary Care Clinic Sites Explained by Co-variates
