# Supplementary material for: Factors Associated With Penicillin Allergy Labels in Electronic Health Records of Children in 2 Large US Pediatric Primary Care Networks
Source: JAMA Netw Open. 2022 Mar 14;5(3):e222117. doi: 10.1001/jamanetworkopen.2022.2117 (PMC9907342; doi:10.1001/jamanetworkopen.2022.2117)
Supplement: Supplement. — eFigure 1. Proportion of Children Labeled as Penicillin Allergic by Age in a Sub-population of Children Remaining in the Birth Cohort Greater Than 4 Years eFigure 2. Proportion of Children Labeled as Penicillin Allergic by the Total Number of Penicillin Prescriptions They Received Prior to Penicillin Allergy Label Placement eTable 1. Proportion of Children Receiving Primary Care Penicillin Prescriptions Before Two Years of Age by Race and Ethnicity eTable 2. Patient Demographics Among Penicillin Allergic and Non-Allergic Children by Primary Care Network eTable 3. Percent of Variation in Penicillin Allergy Label Prevalence Among the Primary Care Clinic Sites Explained by Co-variates [file jamanetwopen-e222117-s001.pdf]

Supplemental Online Content

Taylor MG, Joerger T, Li Y, et al. Factors associated with penicillin allergy labels in electronic health records of children in 2 large US pediatric primary care networks. *JAMA Netw Open.* 2022;5(3):e222117. doi:10.1001/jamanetworkopen.2022.2117

eFigure 1. Proportion of Children Labeled as Penicillin Allergic by Age in a Sub-population of Children Remaining in the Birth Cohort Greater Than 4 Years

eFigure 2. Proportion of Children Labeled as Penicillin Allergic by the Total Number of Penicillin Prescriptions They Received Prior to Penicillin Allergy Label Placement

eTable 1. Proportion of Children Receiving Primary Care Penicillin Prescriptions Before Two Years of Age by Race and Ethnicity

eTable 2. Patient Demographics Among Penicillin Allergic and Non-Allergic Children by Primary Care Network

eTable 3. Percent of Variation in Penicillin Allergy Label Prevalence Among the Primary Care Clinic Sites Explained by Co-variates

This supplemental material has been provided by the authors to give readers additional information about their work.

© 2022 Taylor MG et al. *JAMA Network Open.*

Supplemental Figures & Tables

eFigure 1. Proportion of children labeled as penicillin allergic by age in a sub-population of children remaining in the birth cohort greater than 4 years. Bar graph (left axis) proportion of children labeled in each age group. Line graph (right axis) cumulative proportion of children labeled as penicillin allergic in the birth cohort. Error bars= 95 % confidence intervals.

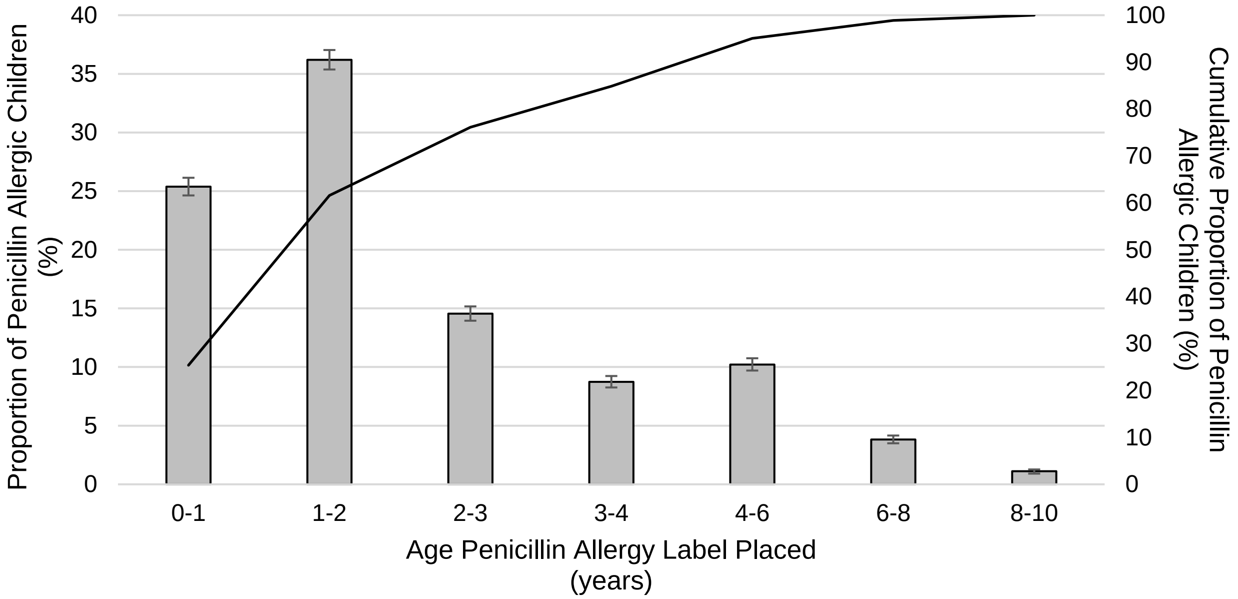

© 2022 Taylor MG et al. *JAMA Network Open.*

eFigure 2. Proportion of children labeled as penicillin allergic by the total number of penicillin prescriptions they received prior to penicillin allergy label placement. Penicillin prescriptions were evaluated from all healthcare sites (including primary care centers, urgent cares, emergency centers, hospitals, and subspecialty clinics).

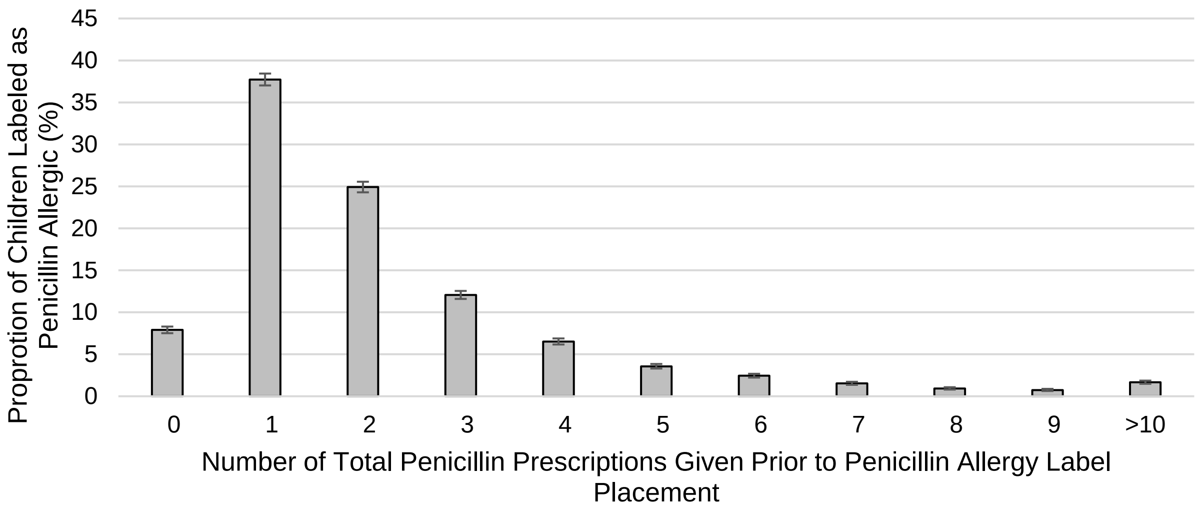

© 2022 Taylor MG et al. *JAMA Network Open.*

eTable 1. Proportion of children receiving primary care penicillin prescriptions before two years of age by race and ethnicity.

Primary care penicillin prescription given by 2 years age	Non-Hispanic White (N = 148,534)	Hispanic (N = 72,831)	Non-Hispanic Black (N = 59,598)	Asian/Pacific Islander (N = 21,081)	Chi2 p value
No	57,323 (38.6 %)	33,695 (46.3 %)	N = 34,365 (57.7 %)	N = 11,544 (54.8 %)	<0.001
Yes	91,211 (61.4 %)	39,136 (53.7 %)	25,233 (42.3 %)	9,537 (45.2 %)	

*Data from children identified as other/missing race/ethnicity not shown.

© 2022 Taylor MG et al. *JAMA Network Open.*

eTable 2. Patient demographics among penicillin allergic and non-allergic children by primary care network. IQR = interquartile range

	**Texas Children's Pediatrics n = 206,451**		**Children's Hospital of Philadelphia n = 128,014**	
**Penicillin Allergy Status**	**Penicillin Allergic n = 10,434 (5.1 %)**	**Penicillin Non-Allergic n = 196,017 (94.9 %)**	**Penicillin Allergic n = 7,581 (5.9 %)**	**Penicillin Non-Allergic n = 120,433 (94.1 %)**
**Sex**				
Female	4,827 (46.3 %)	96,774 (49.4 %)	3,504 (46.2 %)	59,068 (49.1 %)
Male	5,607 (53.7 %)	99,243 (50.6 %)	4,077 (53.8 %)	61,365 (50.9 %)
**Ethnicity and Race**				
Asian or Pacific Islander	687 (6.7 %)	13,859 (7.1 %)	322 (4.2 %)	6,213 (5.2 %)
Hispanic	2,650 (25.4 %)	57,651 (29.4 %)	584 (7.7 %)	11,946 (9.9 %)
Non-Hispanic Black	791 (7.6 %)	25,599 (13.1 %)	860 (11.3 %)	32,348 (26.9 %)
Non-Hispanic White	5,665 (54.2 %)	80,343 (41.0 %)	5,024 (66.3 %)	57,502 (47.7 %)
**Primary Language**				
English	9,984 (95.7 %)	183,994 (93.9 %)	7,126 (94.0 %)	108,724 (90.3 %)
Spanish	255 (2.4 %)	6,256 (3.2 %)	117 (1.5 %)	2,298 (1.9 %)
Other	41 (0.4 %)	1,051 (0.5 %)	67 (0.9 %)	1,340 (1.1 %)
Missing	154 (1.5 %)	4,715 (2.4 %)	271 (3.6 %)	8,071 (6.7 %)
**Government Insurance**	2,450 (23.5 %)	61,697 (31.5 %)	2,420 (31.9 %)	53,421 (44.4 %)
**Chronic Condition**	819 (7.9 %)	13,898 (7.1 %)	500 (7.7 %)	9,354 (7.8 %)
**Median Age in Years (IQR)**	5.1 (3.0, 7.3)	2.8 (1.3, 5.4)	5.2 (2.7, 7.9)	6.7 (4.5, 8.9)

© 2022 Taylor MG et al. *JAMA Network Open.*

eTable 3. Percent of variation in penicillin allergy label prevalence among the primary care clinic sites explained by co-variates.

Co-Variate	Percentage of Primary Clinic Variation Explained by Co-Variate (%)*
Ethnicity and Race	49.9
Age	24.1
Government Insurance	18.2
Chronic Condition	0.6
Primary Language	0.3
Sex	0.1
Healthcare Visits by Two Years	52.8
Primary Care Penicillin Given by Two Years	34.9
Healthcare Communications by Two Years	29.1
All Variables	83.5

*Sum of variation explained by each variable independently will not add up to percentage of variation explained by all of variables together.

© 2022 Taylor MG et al. *JAMA Network Open.*
